# What do diatom indices indicate? Modeling the specific pollution sensitivity index

**DOI:** 10.1007/s11356-024-33115-1

**Published:** 2024-04-05

**Authors:** Saúl Blanco

**Affiliations:** Diatom Laboratory, University of Leon, La Serna 58, 24007 Leon, Spain

**Keywords:** Bioindication, Water quality, Ecological modeling, Biotic indices

## Abstract

Diatoms are commonly used in environmental assessments to detect pollution and eutrophication. The specific pollution sensitivity index (SPI) is one of the most frequently used indices, which assigns scores to diatom taxa based on their sensitivity to pollution. The study analyzed diatom communities in the Duero River basin in Spain to examine the relationship between SPI scores and various limnological variables. A GLM model showed that phosphates, nitrites, and water temperature were the main factors explaining SPI variability. The study also reviews previous results using SPI for water quality monitoring in different world regions, highlighting the role of nutrients in general as major drivers of SPI values worldwide. Overall, the findings reinforce the reliability of SPI as a metric for biological monitoring in various watercourses.

**Supplementary Information:**

The online version contains supplementary material available at 10.1007/s11356-024-33115-1.

## Introduction

Biological indicators offer comprehensive assessments of the highly variable spatial and temporal environmental conditions in streams and rivers. These indicators are crucial components of environmental assessments, aligning with the objectives of many conservation and management programs. In particular, benthic diatoms—the main constituent of river phytobenthos—are commonly used as biological quality elements (BQEs) in surface water monitoring. They have been successfully used to detect eutrophication, organic pollution, and acidification in rivers (Masouras et al. 2021). Diatom-based indices have been widely used for river biomonitoring as an effective tool in assessing water quality and detecting environmental pollution and eutrophication. These indices vary in terms of the number of taxa used for their development, sensitivity values (optima), indicator values (tolerance) assigned to each taxon, and the water quality information they provide, whether it be a general index, trophic index, or organic pollution index. Several diatom-based indices have been developed and validated for this purpose. The diatom-based eutrophication/pollution index (EPI-D) (Torrisi & Dell’Uomo 2006), Biological Diatom Index (IBD) (Prygiel et al. 2002), and Watanabe’s Index (WAT) (Watanabe et al. 1986) have been found to be robust measures of water quality and have been used in large rivers (Tan et al. 2021). Recent advances in diatom biomonitoring include the development of trait-based indices, DNA sequencing, and predictive modeling, which could provide more accurate results in water quality assessments (Dalu et al. 2020).

Diatoms, with their shorter generation times compared to fish and macro-invertebrates, exhibit rapid responses to environmental changes, making them valuable as early warning indicators for detecting pollution increases and assessing habitat restoration success (Mbao et al. 2022). Moreover, the relatively low costs associated with sampling and analysis in comparison to other organisms make diatoms an attractive choice. Their ease of collection over extended periods further supports their utility. Consequently, the study of diatoms has become an integral component of monitoring and assessment programs worldwide. Diatoms possess high local and regional diversity, playing a pivotal role in freshwater biodiversity, particularly in streams, and demonstrate relatively strict environmental preferences, establishing a strong connection between community composition and the surrounding environment. These variations in species composition offer an integrated approach to reflect changes in water quality, surpassing traditional chemical sampling methods. Diatoms offer multiple advantages as bioindicators, thanks to their ubiquity and adaptability to diverse aquatic conditions, the ability of benthic communities to integrate water quality variations, straightforward sampling and preparation methods, indefinite preservation of preparations, and the potential for species identification through taxonomic guides with proper training (Taylor et al. 2007b; Soininen 2007; Feio et al. 2009; Venkatachalapathy and Karthikeyan 2015).

Among the available autecological metrics, the SPI (specific pollution sensitivity) index (Cemagref 1982) is one of the most frequently used diatom-based indices in European and non-European countries. SPI is an ‘autecological’ index, which utilizes the relative abundance of species in assemblages along with their ecological preferences, sensitivities, or tolerances, and these have been developed as powerful tools for inferring environmental conditions in ecosystems. Early monitoring studies demonstrated the effectiveness of autecological indices, particularly focusing on diatom diversity as a general indicator of river health. Diatom-based autecological indices hold significant effectiveness in stream and river assessments due to their capacity to provide comprehensive characterizations of physical and chemical conditions based on a single assay of diatom species composition. This approach offers a valuable means of inferring pollution levels and assessing environmental quality in aquatic ecosystems, making it a valuable tool for ecological monitoring and management (Stevenson et al. 1999; Venkatachalapathy and Karthikeyan 2015). Besides, SPI is considered a “reference” index to evaluate the applicability of new methods because i) it is based on the autecological parameters of virtually all the species potentially present in a sample (28,646 taxa considered as of late 2023, this list being constantly revised and updated) (Tan et al. 2017), ii) this index yields minimal residuals in the correlation analyses relating to nutrients (Álvarez-Blanco et al. 2013), and iii) SPI allows stream biomonitoring throughout the year without the interference of the natural temporal variability of diatom communities (Elias et al. 2012). Despite being an index originally designed from river samples obtained in Central Europe, it is routinely employed successfully throughout the world (Triest et al. 2012), including lentic habitats (Soeprobowati et al. 2023) and even edaphic environments (Foets et al. 2020). Its use is mandatory for the establishment of the ecological status of water bodies in several European countries.

Different biomonitoring methods based on diatom communities may produce contrasting assessments due to differences in their sensitivity to various types of pressures (Blanco et al. 2007; Feio et al. 2009), but there is a need for metrics that can provide information on specific aspects of biological quality (Monaghan 2016). In this regard, SPI is known to provide a realistic assessment of water quality, integrating organic pollution, salinity, and eutrophication (Prygiel and Coste 1993; Schneider et al. 2013). Despite this, little effort has been made to gain a better understanding of how the component dimensions of biotic indices influence index performance (Monaghan 2016). For instance, whereas most indices are well calibrated on phosphorus concentrations, the influence of nitrogen or the interaction with pH is largely unknown (Schneider et al. 2013). There is a need for metrics that can provide information on specific aspects of biological quality to clearly communicate the information provided by their summarized numerical value (Monaghan 2016). Previous efforts to modelize the response of diatom metrics (e.g., SPI) to limnological variables (de la Rey et al. 2008; Novais et al. 2014; Tan et al. 2017) were based on limited datasets and/or spatiotemporal scopes. This paper addresses this question by analyzing benthic diatom communities collected in the largest Iberian basin over a span of 3 years, testing previous assumptions that SPI mainly mirrors nutrient concentrations in rivers.

## Materials and methods

### Study area

**Fig. 1:**
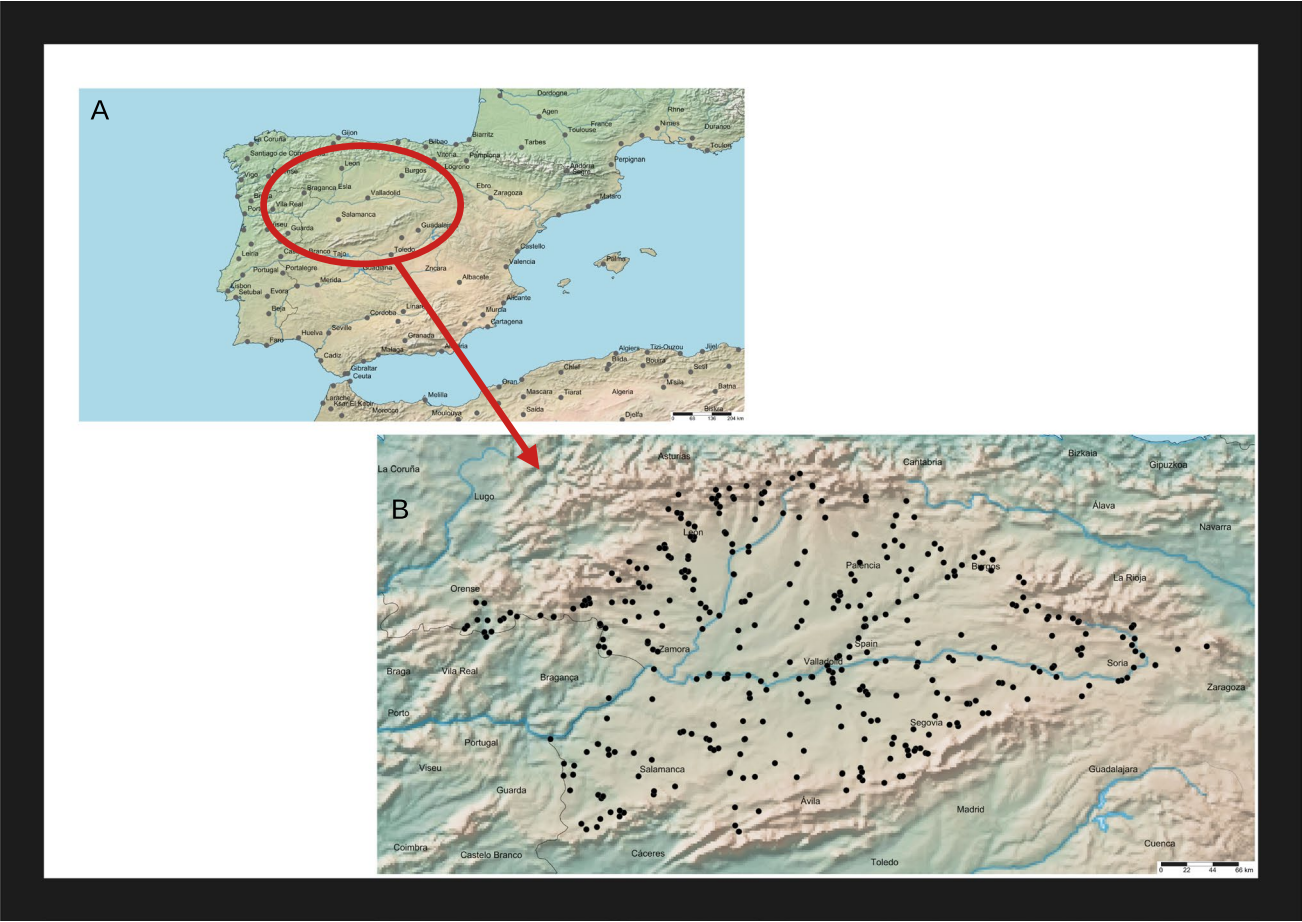
Map showing sampling points in the Duero River basin (NW Iberian Peninsula)

The Duero Basin (97,290 km^2^) is the largest hydrographical basin on the Iberian Peninsula (40–43° N, 1.5–7.5° W, Fig. 1). This study was carried out in the Spanish part of the basin (78,952 km^2^). From a geological point of view, this basin consists of a plateau mostly formed by tertiary and quaternary (alluvial and colluvial) materials. High-relief mountains composed of igneous and metamorphic rocks of Paleozoic age (mainly to the south and west) and siliclastic and carbonate rocks of Mesozoic age (mainly to the East) bound the basin. Most of the basin is situated under Mediterranean bioclimate, here characterized by a strong continental character, with dry summers and cold winters. The mean annual precipitation is 625 mm, concentrated in autumn and winter, whereas there is a pronounced summer precipitation deficit. A network of 80 large reservoirs regulates the flow in the main tributaries of the Duero (Álvarez-Blanco et al. 2011, 2013).

### Sampling and laboratory analyses

A total of 469 samples from 371 stations spread across 225 different watercourses were collected during summers 2007, 2008, and 2009. Sites were selected from the Water Quality Surveillance Network of the Duero Basin Authority (CHD) (Fig. 1). Water chemical variables were provided by the CHD automatic water sampling stations at each site (Table 1). Supplied data consisted of regularly (from hourly to weekly) recorded values, and the measurements corresponding to the closest moment previous to diatom sampling were used. Sampling sites spread randomly throughout the whole basin during all the surveys. Epilithic diatom samples were collected and processed following European standards (Standardization EC 2003). Permanent microscopic slides were obtained and diatom taxa were identified and counted according to European standards (Standardization EC 2004) and usual taxonomic references (Hofmann et al. 2011 and references therein). SPI was calculated using OMNIDIA software ver. 6.1.7 (Lecointe et al. 1993). SPI scores, ranging from 1 to 20, are the average relative abundances of the diatom taxa present in a community, multiplied by their respective sensitivity values—*S*, ranging from 1 (species indicative of very polluted waters) to 5 (species typical of pristine habitats)—weighted by their respective indicator values (*V*, ranging from 1 to 3, with ubiquitous taxa having a value of 1 and the very specific taxa having a value of 3) (Ector and Rimet 2005; Feio et al. 2009), that is,

**Table 1:** Limnological variables measured in the sampling locations. Detailed information available at the Duero Basin Authority Database www.chduero.es

Variable	Median (range)
Alkalinity (ppm)	86.5 (3.0–392.9)
Ammonia (ppm)	0.052 (0.005–7.573)
BOD_5_ (ppm)	1 (1–9)
Conductivity (µS cm^−1^)	218 (10–2160)
Nitrates (ppm)	1.99 (0.01–52.20)
Nitrites (ppm)	0.022 (0.002–1.898)
NTK (ppm)	1.74 (0.33–38.00)
O_2_ (ppm)	8.4 (1.1–17.5)
pH	7.9 (5.3–11.2)
Phosphates (ppm)	0.090 (0.005–12.045)
T (°C)	14.4 (6.0–24.7)
TSS (ppm)	7.9 (0.3–615.7)

$${\text{SPI}}=\frac{\sum A\cdot S\cdot V}{\sum A\cdot V}$$

Water quality of a given site can be then classified according to the resulting SPI score as bad (1–5), poor [5–9], moderate (9–13), good (13–17), or high quality (17–20). The sensitivity and indicator values were derived from multivariate analyses on diatom and chemical data collected in France, although this metric is routinely employed worldwide.

In order to compare our results with those published in former studies, a literature survey was carried out to find reported correlations between SPI and abiotic parameters. A total of 30 papers were identified (Fig. 4), of which the following information was extracted: *R*^2^ values, geographic coordinates (latitude and longitude of the centroid), and sample size. In cases where regression analysis were performed, the coefficients (*β*) of these regressions were also considered.

### Statistical analysis

To assess the independent effects of limnological predictors on SPI scores, we conducted a generalized linear model analysis (GLM) with an identity link function and a Poisson distribution. GLM is used here to examine how quantitative independent parameters affect a dependent variable with a non-normal distribution. The use of the identity link indicate that the explanatory variables are used to predict the expected value of the untransformed response variable. The selection of independent variables was based on a ‘best subsets’ routine, a method that systematically explores all potential combinations of predictor variables to identify the subset that yields the best-fitting regression model according to a specified criterion. About 50% of the data were randomly selected for cross-validation. To compare the performance of the various generated models, we utilized Akaike’s Information Criterion. Finally, a confusion matrix was computed to contrast observed and expected classifications of sites into water quality categories, this matrix tested against the null hypothesis that both classifications are unrelated, using a Chi-squared test and the Kappa statistic. Statistical analyses were conducted using STATISTICA v. 10 (Statsoft 2012).

## Results

### Biotic/abiotic data and SPI scores

**Fig. 2:**
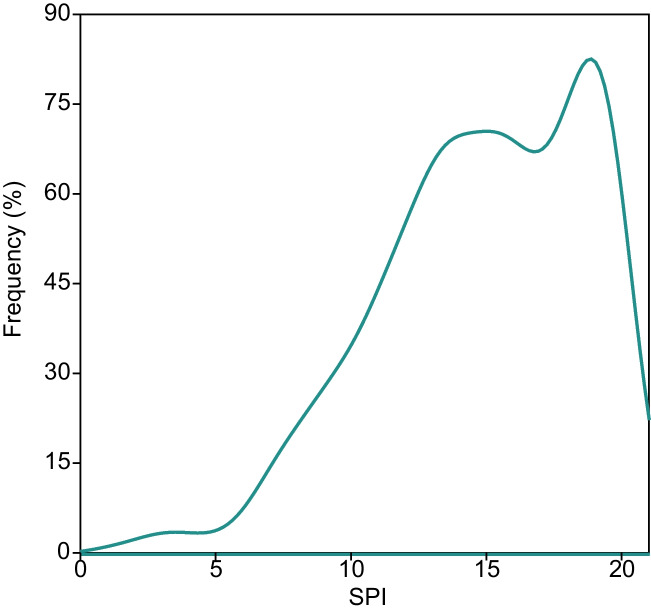
Histogram of SPI values. Data fitted by a kernel smoothing distribution

Limnological variables measured in the sampling sites are summarized on Table 1. The study area covered a wide range of different ecological conditions in terms of electrolyte concentrations (from 9.8 to 2160.0 µS cm^−1^) and nutrient levels (phosphate concentrations ranging from 0.00 to 12.05 ppm). Most sites can be considered circumneutral. Concerning biotic data, a total of *ca.* 2·10^5^ diatom individuals were counted and identified to species or subspecific levels. Diatom communities inhabiting sampling locations consisted on 744 different taxa (species or subspecific level), with a notably large global diversity (Whittaker’s *β* = 28.2). The most widespread and abundant species were the cosmopolitan, eurioic *Achnanthidium minutissimum* (17.7% relative abundance of all counted valves), followed by the oligotrophilous alkalibiont *Achnanthidium pyrenaicum* (11.3%). In general, epilithic diatom assemblages were dominated by species belonging to the genera *Nitzschia*, *Navicula* and *Gomphonema* (78, 72, and 51 species, respectively,). Additional floristic and ecological data concerning these sampling surveys have been published elsewhere (Blanco et al. 2007, 2008; Blanco and Bécares 2010; Álvarez-Blanco et al. 2011, 2013). SPI scores ranked from 1 to 20, covering the whole spectrum of this metric. Most sites reached good or high water quality statuses (SPI ≥ 13, Fig. 2).

### Model building

**Fig. 3:**
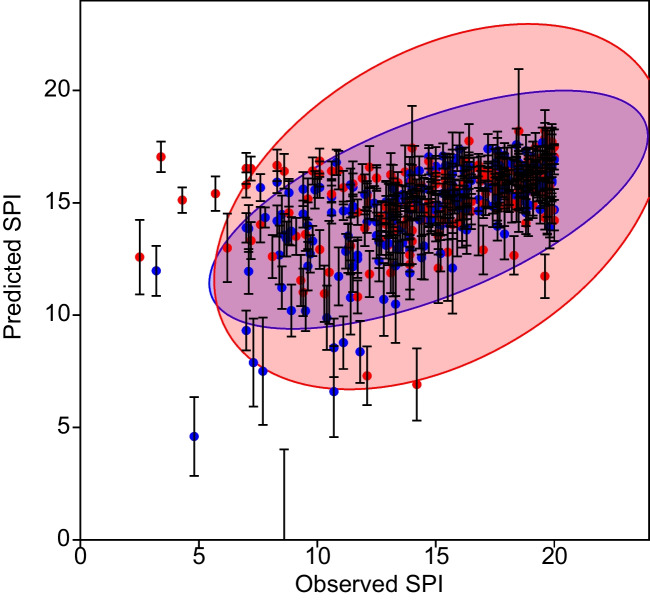
Observed *vs.* predicted SPI values in the training (blue) and cross-validation (red) sets. Error bars denote ± 1 SD. Data fitted to 95% confidence ellipses

**Fig. 4:**
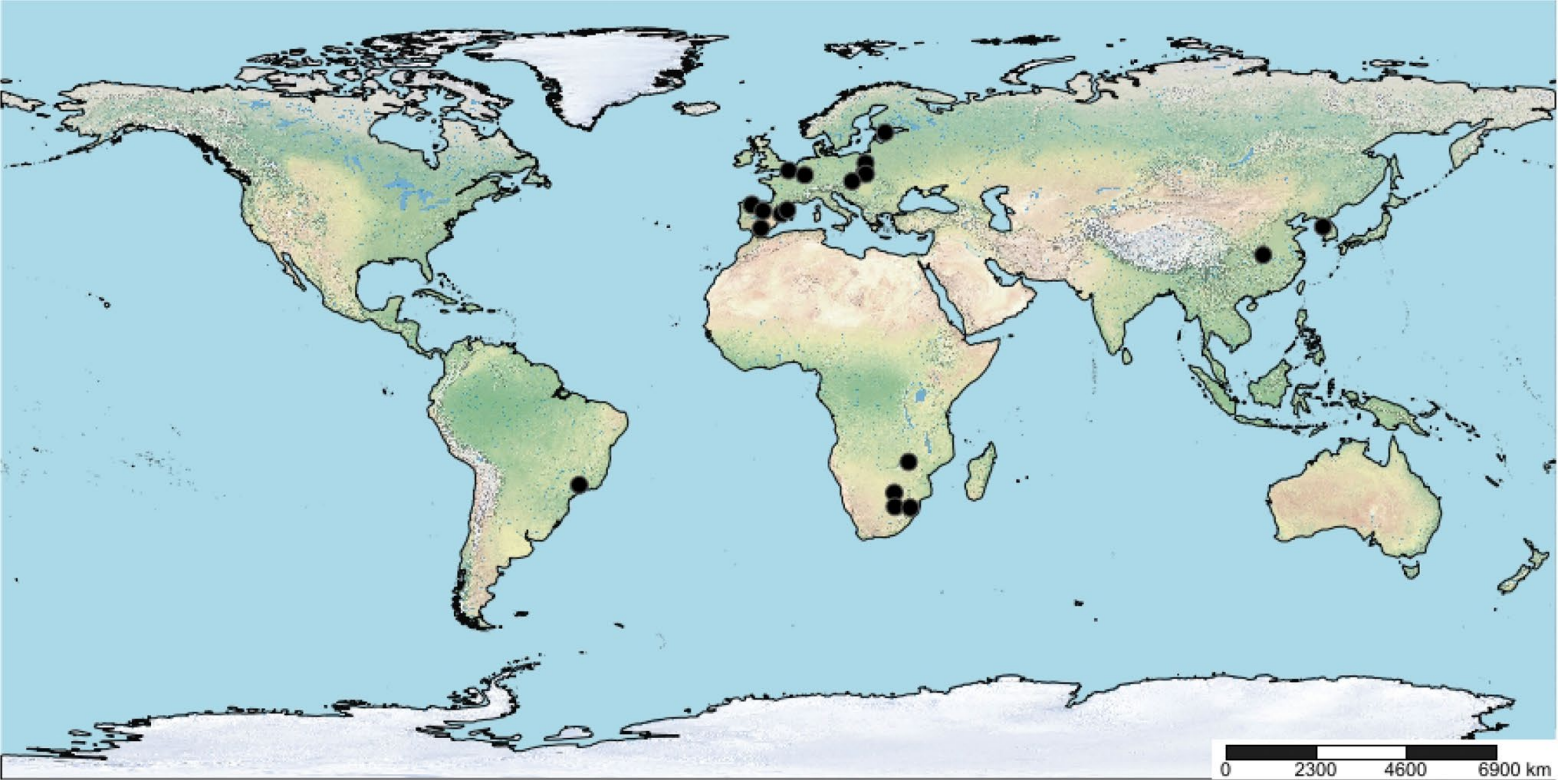
World map showing the location of previous studies using SPI

Among the *ca.* 200 different combinations tested, the GLM model with a ‘best subset’ of predictors consisting on nitrites (likelihood ratio test *p* = 0.006), phosphates (*p* = 0.001), and temperature (*p* = 0.007) achieved the lowest AIC score. Model parameters for the significant variables were estimated as follows: − 8.18 ± 2.94 for nitrites, − 1.94 ± 0.57 for phosphates, and − 0.22 ± 0.08 for temperature (Table 2). Observed and model-predicted SPI values correlated significantly both in the training and the cross-validation sets (*p* < 0.001 in both cases, Figs. 3 and 4). Table 3 presents the confusion matrix resulting from classifying sites according to their observed or predicted SPI scores, this matrix deviating significantly (*χ*^2^ = 143.11, *p* < 0.001) from a lack of correspondence between observed and predicted classes. Kappa statistic with quadratic weighting (0.70) confirmed a ‘substantial’ agreement between both classifications according to the criteria of Landis and Koch (Landis and Koch 1977).

**Table 2:** Model parameters for the abiotic predictors explored in the study

	*p*	Chi-square	Log-likelihood	Wald
Intercept	0.00		− 304.85	18.17
Alkalinity	0.51	0.45	− 266.14	0.43
Ammonia	0.12	2.36	− 267.09	2.48
Conductivity	0.54	0.40	− 266.11	0.38
BOD_5_	0.98	0.00	− 265.91	0.00
Nitrates	0.49	0.48	− 266.15	0.47
Nitrites	0.01	7.67	− 269.75	7.74
NTK	0.97	0.00	− 265.91	0.00
O_2_	0.65	0.21	− 266.01	0.21
pH	0.43	0.63	− 266.22	0.63
Phosphates	0.00	11.49	− 271.66	11.71
TSS	0.28	1.22	− 266.52	1.18
T	0.01	7.33	− 269.58	7.10

**Table 3:** Confusion matrix showing the classification of sites (water quality) based on their observed and predicted SPI scores

		Predicted				
		High	Good	Moderate	Poor	Bad
Observed	Bad	1	1	2	0	2
	Poor	0	20	7	2	2
	Moderate	0	51	20	5	1
	God	5	136	14	1	0
	High	20	129	3	0	0

**Fig. 5:**
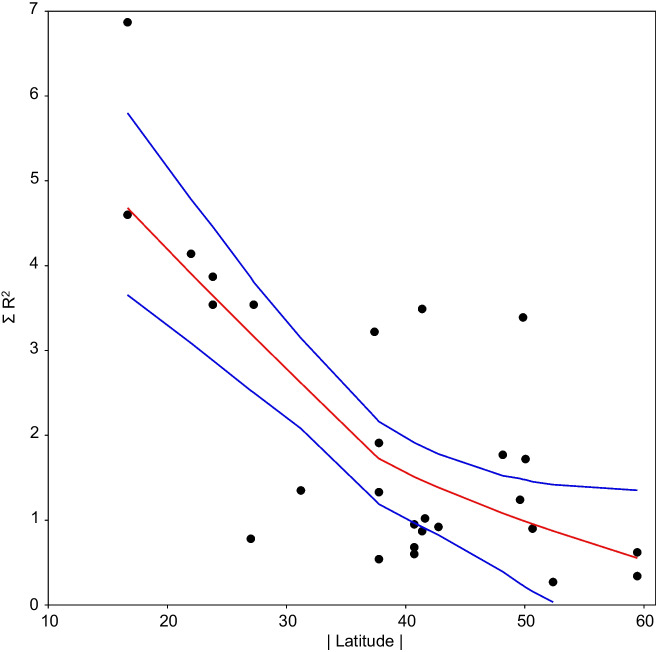

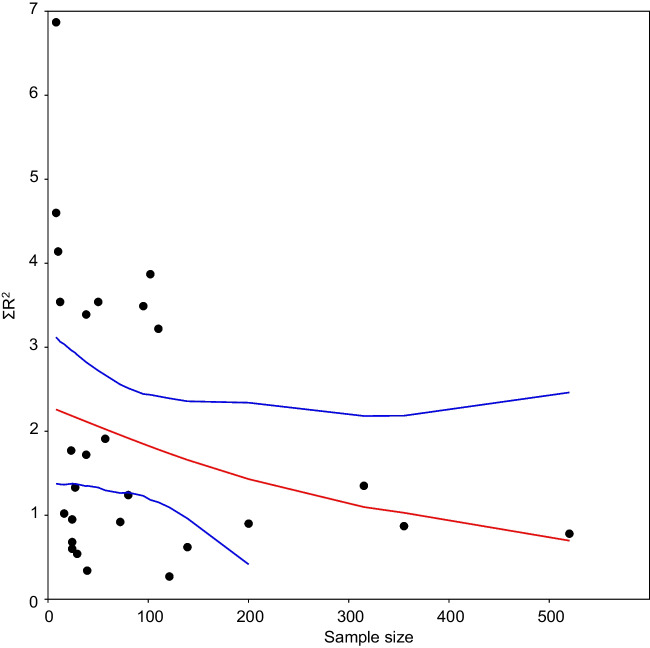
Cumulative correlation coefficient of SPI with respect to abiotic factors, as reported in the literature (*n* = 30). **a** Relationship between Σ*R*^2^ and latitude. **b** Relationship between Σ*R*^2^ and sample size. Data fitted to LOESS smoothers ± 95% bootstrap confidence bands

Concerning the literature survey performed, most of analyzed papers (70%) detected conductivity as a major driver of SPI values, followed by phosphates (67%) and ammonia (53%). In terms of correlation values reported, there was a significant decay in *R*^2^ values along a latitudinal gradient (Fig. 5a). Noticeably, cumulative *R*^2^ values were lower in studies with large sample sizes (Fig. 5b). Some of these studies included also regression models relating SPI with limnological predictors, the corresponding *β* parameters for the variables also considered in our study are gathered on Table 4.

**Table 4:** Regression models proposed for the SPI. *β* values shown

References	(De La Rey et al. 2004)	(Tan et al. 2013)	(Novais et al. 2014)	(Tan et al. 2017)	(De la Rey et al. 2008)	This study
*N*	12	63	92	34	12	469
Alkalinity			− 0.008			
Conductivity				− 0.020		
DO			− 0.024			
Nitrites						− 8.18
pH	0.428				0.305	
Phosphates					0.368	− 1.94
Temperature						0.22
Turbidity	− 0.238					
Whole model *R*^2^	0.990	0.494	0.386	0.820	0.796	0.68

Table S1. Checklist of diatom taxa found in the sampling locations

## Discussion

Analyzed literature confirms that diatom-based indices are useful for river biomonitoring, but there are challenges in their application. Taylor et al. (2007a) found that diatom indices developed in Europe and elsewhere are useful in South Africa to indicate water quality, but a diatom index unique to South Africa including endemic species will have to be formulated. Similarly, Qu et al. (2014) questioned the transferability of European diatom-based indices to other rivers and geographic locations, but found that the European diatom index SPI was applicable to the Taizi River in northeastern China. Such studies suggest that diatom-based indices can be useful for river biomonitoring, but their applicability varies depending on the catchment setting, river types, and the combination of indices used, with some metrics showing better correlation with certain environmental variables. As explained by Stenger-Kovács et al. (2007), variations in inferred water quality across different ecoregions can be quite substantial, primarily due to i) changes in the autecological preferences of dominant taxa (Álvarez-Blanco et al. 2011), ii) disparities in the extent (floristic coverage) of the databases, including variations in sample size, and iii) taxonomic identification discrepancies. Our results show actually a decay in SPI performance along a latitudinal gradient, showing that this metric may reflect water conditions in tropical areas even better than in mid-latitudes where the index was originally developed. Additionally, water quality assessments based on relatively low sample sizes may work better than in large areas (Fig. 5b) probably due to the intrinsic larger variability within each abiotic parameter in this case.

Ecological assessment methods have shown that diatom indices exhibit stronger correlations with water chemical variables, while macroinvertebrate-based approaches tend to be more sensitive to changes affecting structural parameters (Blanco et al. 2007). Numerous stream diatom studies, as inferred from our literature survey, have highlighted major ion concentrations as primary determinants of diatom distribution, with conductivity, pH, alkalinity, and calcium concentration emerging as key environmental gradients. Additionally, trophic status indicators such as total phosphorus, chlorophyll, total nitrogen, and inorganic nutrient concentrations have been identified as significant environmental correlates of lotic diatoms. Therefore, river diatom community composition is closely associated with water chemical properties, while physical in-stream factors have a relatively smaller impact on community composition. Diatom indices, reflecting an integration of the water quality variables they have been exposed to over a specific period, are particularly informative in this river system, although this relationship may vary in shallower, faster-flowing streams with localized pollution sources (Taylor et al. 2007b; Sgro et al. 2007; Soininen 2007; Qu et al. 2014).

SPI is regarded as the most adequate index for biological monitoring in a wide variety of watercourses (Prygiel et al. 1999; Blanco et al. 2007; Hlúbiková et al. 2007). Overall, the main drivers of SPI values are a combination of environmental variables and spatial factors. Comparative studies on the performance of different metrics are usually carried out assessing the correlation between metrics scores and limnological variables. In France, SPI shows significant correlations with ionic strength (expressed by chloride, sulfate, and conductivity) and eutrophication (expressed by chlorophyll and nitrate) (Prygiel and Coste 1993). In Poland, SPI significantly correlated with organic load expressed by COD and BOD_5_, DO, ionic composition, and trophic level expressed by inorganic N and P concentrations (Kwandrans et al. 1998). However, other studies (Tison et al. 2008) suggest that SPI is mostly driven by organic matter enrichment. Despite this index originally developed to detect general pollution, it is usually strongly correlated with both nutrients and organic pollution (Kwandrans et al. 1998; Schneider et al. 2013), and this may be merely reflecting the frequent collinearity between these stressors that cannot be disentangled using simple correlation analysis. In our analysis, the likelihood ratio test used to assess the significance of abiotic factors tests the increment in the log-likelihood attributable to each predictor separately—while controlling for all other effects—and our results based on such GLM modeling show that, contrary to nutrients, BOD_5_ had a negligible independent effect on SPI scores. This ability to separate nutrient-rich waters from those that are organically polluted ones is considered an important feature in diatom metrics (Kelly 1998a, b).

Comparative studies based on correlation analysis reveal that SPI is the most suitable metric for conducting biological monitoring in other regions (Kelly et al. 1995, 2001; Prygiel et al. 1999; Blanco et al. 2007). For instance, Tan et al. (2017) showed that most of the variation in the SPI was explained by parameters such as electric conductivity or soluble reactive phosphorus. Although it is often difficult to distinguish specific nutrients causing the effect (Bate et al. 2004), our analysis point to the concentration of phosphates and nitrites as the main explanatory factors among nutrients for SPI variability. Interestingly, the majority of diatom indices are calibrated based on phosphorus concentrations, and there is limited understanding of how nitrogen affects these indices (Schneider et al. 2013). Diatoms are also known to be extremely sensitive to pH and salinity (Soininen 2007; Venkatachalapathy and Karthikeyan 2015), but these parameters were discarded in our SPI statistical model evidencing that this metric may not capture all potential stressors in lotic habitats. It is known that SPI can even fail to reflect hydrochemical characteristics in springs (Prygiel et al. 1999) or fast-flowing streams subject to point source impacts (Taylor et al. 2007b).

Previous regression models proposed for the SPI (Table 4) show that there is not a consistent set of predictors accounting for SPI values worldwide. Our results demonstrate, however, that a simple model accounting for phosphates and nitrites concentrations, together with water temperature, may reconstruct accurately SPI scores at a water basin scale. As aforementioned, the role of phosphates as major drivers of SPI and other diatom indices is widely reported, but the significant contribution of temperature and nitrites (and not other nutrients) is striking. In Luxembourg, SPI also correlated with nitrites and temperature (Hlúbiková et al. 2014), despite nitrates and ammonia were also measured, and Zgrundo and Bogaczewicz-Adamczak (2004) found that the diatom index EPI-D was mostly affected by nitrites. Nitrites are the transitional, toxic forms of nitrogen under anaerobic conditions, and certain extremely impaired locations in our dataset reached concentrations up to 2 ppm (Table 1). In these samples, the link between nitrites and SPI scores may be related to the dominance of species indicating very bad conditions such as *Nitzschia palea* or *Nitzschia capitellata*, known to correlate highly with nitrites (Benhassane et al. 2020). Finally, the dependence of diatom-based metrics on water temperature has been assessed by other authors (Prygiel and Coste 1993; Taylor et al. 2007b). On the contrary, Elias et al. (Elias et al. 2012) confirmed the influence of temperature on diatom communities’ structure but not on the results of the SPI. In our case, seasonality can be discarded since sample collection took place during summer in all cases, so that water temperature exerts a certain effect on water quality (as measured by SPI) by itself. This variable acts as a surrogate of other underlying physical and chemical factors, which in turn affect the structure and composition diatom assemblages (Jakovljević et al. 2016; Çetin and Demir 2019) and even diatom guilds (Hlúbiková et al. 2014; Trábert et al. 2017).

## Conclusions

Our research findings reinforce the reliability of the specific pollution sensitivity index (SPI) as a reliable metric for biological monitoring in diverse watercourses. While correlations with various limnological parameters have been observed in different regions, our analysis highlights the importance of phosphates and nitrites, in conjunction with water temperature, in explaining SPI variability at a water basin scale. This underscores the role of nutrients as a major driver of SPI and other diatom indices.

In summary, our study demonstrates that a straightforward model considering phosphates and nitrites concentrations, along with water temperature, can effectively reconstruct SPI scores. Nevertheless, the complexity of diatom responses to environmental variables and the potential for regional variation remind us that diatom-based indices should be applied with careful consideration of local conditions and, when necessary, adapted to specific regions or river types to ensure accurate water quality assessment. In this regard, DNA metabarcoding and high-throughput sequencing are being applied to diatom biomonitoring, improving data quantity and resolution (Maitland et al. 2020). Overall, diatom-based indices, either based on DNA metabarcoding techniques or traditional microscopy-based methods, have advanced the state-of-the-art in river biomonitoring using diatoms as indicators of water quality (Goldenberg-Vilar et al. 2020).

### Supplementary Information

Below is the link to the electronic supplementary material.

Supplementary file1 (CSV 34.2 KB)

## References

[CR1] Álvarez-Blanco I, Cejudo-Figueiras C, Bécares E, Blanco S (2011). Spatiotemporal changes in diatom ecological profiles: implications for biomonitoring. Limnology.

[CR2] Álvarez-Blanco I, Blanco S, Cejudo-Figueiras C, Bécares E (2013). The Duero Diatom Index (DDI) for river water quality assessment in NW Spain: design and validation. Environ Monit Assess.

[CR3] Bate G, Smailes P, Adams J (2004). A water quality index for use with diatoms in the assessment of rivers. Water SA.

[CR4] Benhassane L, Oubraim S, Mounjid J (2020). Monitoring impacts of human activities on Bouskoura stream (Periurban of Casablanca, Morocco): 3. Bio-ecology of epilithic diatoms (first results). Nat Environ Pollut Technol.

[CR5] Blanco S, Bécares E (2010). Are biotic indices sensitive to river toxicants? A comparison of metrics based on diatoms and macro-invertebrates. Chemosphere.

[CR6] Blanco S, Ector L, Huck V (2008). Diatom assemblages and water quality assessment in the Duero Basin (nw Spain). Belg J Bot.

[CR7] Blanco S, Bécares E, Cauchie H-M, et al (2007) Comparison of biotic indices for water quality diagnosis in the Duero Basin (Spain). Large Rivers 267–286

[CR8] Cemagref (1982) Étude des méthodes biologiques quantitative d’appréciation de la qualité des eaux. Rapport Division Qualité des Eaux Lyon—Agence financière de Bassin Rhône-Méditerranée-Corse: Pierre-Bénite 218:

[CR9] Çetin T, Demir N (2019). The use of phytobenthos for the ecological status assessment in Upper Sakarya Basin, Turkey. Appl Ecol Environ Res.

[CR10] Dalu T, Cuthbert RN, Taylor JC (2020). Benthic diatom-based indices and isotopic biomonitoring of nitrogen pollution in a warm temperate Austral river system. Sci Total Environ.

[CR11] De La Rey PA, Taylor JC, Laas A (2004). Determining the possible application value of diatoms as indicators of general water quality : a comparison with SASS 5. Water SA.

[CR12] De la Rey PA, Roux H, van Rensburg L, Vosloo A (2008). On the use of diatom-based biological monitoring part 2: a comparison of the response of SASS 5 and diatom indices to water quality and habitat variation. Water SA.

[CR13] Ector L, Rimet F (2005). Using bioindicators to assess rivers in Europe: an overview. Modell Commun Struct Freshwater Ecosyst.

[CR14] Elias CL, Vieira N, Feio MJ, Almeida SFP (2012). Can season interfere with diatom ecological quality assessment?. Hydrobiologia.

[CR15] Feio MJ, Almeida SFP, Craveiro SC, Calado AJ (2009). A comparison between biotic indices and predictive models in stream water quality assessment based on benthic diatom communities. Ecol Ind.

[CR16] Foets J, Wetzel CE, Teuling AJ, Pfister L (2020). Temporal and spatial variability of terrestrial diatoms at the catchment scale: controls on communities. PeerJ.

[CR17] Goldenberg-Vilar A, Álvarez-Troncoso R, Roldán V, Blanco S, Cristóbal G, Blanco S, Bueno G (2020). Water Quality Assessment. Modern trends in diatom identification: fundamentals and applications.

[CR18] Hlúbiková D, Novais MH, Dohet A (2014). Effect of riparian vegetation on diatom assemblages in headwater streams under different land uses. Sci Total Environ.

[CR19] Hlúbiková D, Hindáková A, Haviar M, Miettinen J (2007). Application of diatom water quality indices in influenced and non-influenced sites of Slovak rivers (Central Europe). Large Rivers.

[CR20] Hofmann G, Werum M, Lange-Bertalot H (2011) Diatomeen im Süßwasser-Benthos von Mitteleuropa: Bestimmungsflora Kieselalgen für die ökologische Praxis; über 700 der häufigsten Arten und ihrer Ökologie. Gantner

[CR21] Jakovljević OS, Popović SS, Vidaković DP (2016). The application of benthic diatoms in water quality assessment (Mlava River, Serbia). Acta Bot Croat.

[CR22] Kelly M (1998). Use of The trophic diatom index to eutrophication in rivers Monıtor. Water Res.

[CR23] Kelly MG (1998). Use of community-based indices to monitor eutrophication in European rivers. Environ Conserv.

[CR24] Kelly MG, Penny CJ, Whitton BA (1995). Comparative performance of benthic diatom indices used to assess river water quality. Hydrobiologia.

[CR25] Kelly MG, Adams C, Graves AC, Jamieson J, Krokowski J, Lycett EB, ... Wilkins C (2001) The trophic diatom index: a user’s manual. Bristol: Environment Agency, p 135

[CR26] Kwandrans J, Eloranta P, Kawecka B, Wojtan K (1998). Use of benthic diatom communities to evaluate water quality in rivers of southern Poland. J Appl Phycol.

[CR27] Landis JR, Koch GG (1977). The measurement of observer agreement for categorical data. Biometrics.

[CR28] Lecointe C, Coste M, Prygiel J (1993). “Omnidia”: software for taxonomy, calculation of diatom indices and inventories management. Hydrobiologia.

[CR29] Maitland VC, Robinson CV, Porter TM, Hajibabaei M (2020). Freshwater diatom biomonitoring through benthic kick-net metabarcoding. PLoS ONE.

[CR30] Masouras A, Karaouzas I, Dimitriou E (2021). Benthic diatoms in river biomonitoring—present and future perspectives within the water framework directive. Water.

[CR31] Mbao EO, Odinga ES, Nyika J (2022). A bibliometric study on the use of diatoms in water quality monitoring and bioassessment in Africa across 10-year (2012–2022) period. Aquat Sci.

[CR32] Monaghan KA (2016). Four reasons to question the accuracy of a biotic index; the risk of metric bias and the scope to improve accuracy. PLoS ONE.

[CR33] Novais MH, Morais MM, Rosado J (2014). Diatoms of temporary and permanent watercourses in Southern Europe (Portugal). River Res Appl.

[CR34] Prygiel J, Coste M (1993). Utilisation des indices diatomiques pour la mesure de la qualité des eaux du bassin Artois-Picardie : bilan et perspectives. Ann Limnol Int J Limnol.

[CR35] Prygiel J, Carpentier P, Almeida S (2002). Determination of the biological diatom index (IBD NF T 90–354): results of an intercomparison exercise. J Appl Phycol.

[CR36] Prygiel J, Coste M, Bukowska J (1999). Review of the major diatom-based techniques for the quality assessment of rivers - state of the art in Europe. Use Algae Monit Rivers.

[CR37] Qu X, Zhou Y, Zhao R (2014). Are diatom-based indices from Europe suitable for river health assessment in China? A case study from Taizi River, northeastern China. Br J Environ Clim Change.

[CR38] Schneider SC, Kahlert M, Kelly MG (2013). Interactions between pH and nutrients on benthic algae in streams and consequences for ecological status assessment and species richness patterns. Sci Total Environ.

[CR39] Sgro GV, Reavie ED, Kingston JC (2007). A diatom quality index from a diatom-based total phosphorus inference model. Environ Bioindic.

[CR40] Soeprobowati TR, Saraswati TR, Jumari J (2023). Diatom index of Galela Lake, Halmahera, Indonesia in relation to human activities. Int J Environ Sci Technol.

[CR41] Soininen J (2007). Environmental and spatial control of freshwater diatoms—a review. Diatom Res.

[CR42] Standardization EC (2004). Water quality: guidance standard for the identification, enumeration and interpretation of benthic diatom samples from running waters. Eur Standard EN.

[CR43] Standardization EC for (2003) Water quality: guidance standard for the routine sampling and pretreatment of benthic diatoms from rivers. EN 13946

[CR44] Statsoft I (2012) STATISTICA (data analysis software system), version 10.0. Tulsa: StatSoft

[CR45] Stenger-Kovács C, Buczkó K, Hajnal É, Padisák J (2007). Epiphytic, littoral diatoms as bioindicators of shallow lake trophic status: Trophic Diatom Index for Lakes (TDIL) developed in Hungary. Hydrobiologia.

[CR46] Stevenson RJ, Pan Y, Van Dam H (1999). Assessing environmental conditions in rivers and streams with diatoms. The diatoms.

[CR47] Tan X, Sheldon F, Bunn SE, Zhang Q (2013). Using diatom indices for water quality assessment in a subtropical river, China. Environ Sci Pollut Res.

[CR48] Tan X, Zhang Q, Burford MA (2017). Benthic diatom based indices for water quality assessment in two subtropical streams. Front Microbiol.

[CR49] Tan X, Liu Y, Burford MA, Zhang Q (2021). The performance of diatom indices in assessing temporal changes in water quality in a large lowland river ecosystem. River Res Appl.

[CR50] Taylor JC, Prygiel J, Vosloo A (2007). Can diatom-based pollution indices be used for biomonitoring in South Africa? A case study of the Crocodile West and Marico water management area. Hydrobiologia.

[CR51] Taylor JC, Vuuren MJ van, Pieterse AJH (2007b) The application and testing of diatom-based indices in the Vaal and Wilge Rivers, South Africa. Water SA 33. 10.4314/wsa.v33i1.47871

[CR52] Tison J, Giraudel J-L, Coste M (2008). Evaluating the ecological status of rivers using an index of ecological distance: an application to diatom communities. Ecol Ind.

[CR53] Torrisi M, Dell’Uomo A (2006). Biological monitoring of some apennine rivers (central Italy) using the diatom-based eutrophication / pollution index (epi-D) compared to other European diatom indices. Diatom Res.

[CR54] Trábert Z, Kiss KT, Várbíró G (2017). Comparison of the utility of a frequently used diatom index (IPS) and the diatom ecological guilds in the ecological status assessment of large rivers. Fundam Appl Limnol.

[CR55] Triest L, Lung’ayia H, Ndiritu G, Beyene A (2012). Epilithic diatoms as indicators in tropical African rivers (Lake Victoria catchment). Hydrobiologia.

[CR56] Venkatachalapathy R, Karthikeyan P, Kumaraswamy K, Mohanraj R (2015). Application of diatom-based indices for monitoring environmental quality of riverine ecosystems: a review. Ramkumar Mu.

[CR57] Watanabe T, Asai K, Houki A (1986). Numerical estimation to organic pollution of flowing water by using the epilithic diatom assemblage ––- diatom assemblage index ( DAIpo ) ––. Sci Total Environ.

[CR58] Zgrundo A, Bogaczewicz-Adamczak B (2004). Applicability of diatom indices for monitoring water quality in coastal streams in the Gulf of Gdańsk Region, northern Poland. Oceanol Hydrobiol Stud.

